# Valproic Acid Significantly Improves CRISPR/Cas9-Mediated Gene Editing

**DOI:** 10.3390/cells9061447

**Published:** 2020-06-10

**Authors:** Hanseul Park, Jaein Shin, Hwan Choi, Byounggook Cho, Jongpil Kim

**Affiliations:** 1 Department of Biomedical Engineering (BK21 Plus), Dongguk University, Seoul 04620, Korea; phs509@gmail.com (H.P.); sji1124@naver.com (J.S.); tearfate@naver.com (H.C.); whqudrnr9286@gmail.com (B.C.); 2 Laboratory of Cell reprogramming and Gene editing, Department of Chemistry, Dongguk University, Seoul 04620, Korea

**Keywords:** embryo, gene editing, CRISPR/Cas9, valproic acid

## Abstract

The clustered regularly interspaced short palindromic repeats (CRISPR)/Cas9 system has emerged as a powerful technology, with the potential to generate transgenic animals. Particularly, efficient and precise genetic editing with CRISPR/Cas9 offers immense prospects in various biotechnological applications. Here, we report that the histone deacetylase inhibitor valproic acid (VPA) significantly increases the efficiency of CRISPR/Cas9-mediated gene editing in mouse embryonic stem cells and embryos. This effect may be caused through globally enhanced chromatin accessibility, as indicate by histone hyperacetylation. Taken together, our results suggest that VPA can be used to increase the efficacy of CRISPR/Cas9 in generating transgenic systems.

## 1. Introduction

Genome editing is a type of genetic engineering, in which genetic information, DNA, is modified within an organism’s genome [1]. Recently, the clustered regularly interspaced short palindromic repeat (CRISPR)/Cas9 system has emerged as a powerful and precise method for gene inactivation via the formation of small insertions/deletions (InDels) and for specific genome editing via homology-directed recombination [2,3,4,5,6]. The CRISPR/Cas9 system consists of a Cas9 nuclease protein and single-guide RNA (sgRNA), which directs the Cas9 nuclease to the target genomic locus. In particular, its fast and easy application make CRISPR/Cas9-mediated gene editing an ideal technique for generating various transgenic organisms [7,8]. Based on this concept, several studies have demonstrated that the CRISPR/Cas9 system can drive gene disruption, activation, repression, and genome targeting in various organisms [9,10,11]. For example, CRISPR/Cas9-induced precise genome editing has been commonly performed in mice and includes zygote-based microinjection or embryonic stem cell (ESC)-based transfection approaches [12,13,14,15,16]. However, although CRISPR/Cas9 is a promising tool for gene targeting in various biological systems, it is still relatively inefficient in inducing genome editing in several systems [17,18,19]. Additionally, the low efficiency of homology-directed repair (HDR)-mediated genetic recombination hinders the application of this technology in transgenesis [20,21].

Small molecules can be used to control fundamental biological processes, such as survival, self-renewal, and differentiation [22,23]. Accordingly, small-molecule–based chemical approaches may also facilitate transgenic systems. Particularly, such approaches offer distinct advantages in the gene-editing field, since they can be temporally regulated to rapidly inhibit or activate specific targeting processes, and these effects are often reversible. Additionally, the effects of small molecules on the gene editing can be finely tuned by varying the treatment dosage or period. However, to date, few studies have shown the enhancement of small molecule-induced gene editing, especially in embryos [24,25,26,27]. In this regard, reinforcement of the CRISPR/Cas9 system with small molecules can be an ideal strategy for therapeutic gene editing.

In this study, we sought to identify small molecules that enhance CRISPR/Cas9-mediated gene targeting. We found that valproic acid (VPA) could increase the efficiency of Cas9-mediated gene editing in mouse embryonic stem cells (ESCs) and embryos. VPA is a small molecule that has been shown to affect several pathways [28,29,30]. For example, as an epigenetic modifier, it has a profound impact on the chromatin structure through inhibiting histone deacetylases (HDACs), and this effect significantly improves cellular reprogramming efficiency [31]. VPA has also been reported to affect several signaling pathways and regulate the differentiation and proliferation of various cells [32,33]. Since histone acetylation is one of the key epigenetic modifications affecting chromatin packaging, and often associated with an open chromatin configuration, inhibition of histone deacetylases by VPA alters the chromatin structure and makes the DNA more accessible for gene editing. Thus, this study indicates the importance of the chromatin structure for efficient and precise gene editing and suggests that VPA-augmented Cas9 gene editing can be an effective approach for generating transgenic applications.

## 2. Materials and Methods

### 2.1. Preparation of the RNP Complexes and Donor Vector

Recombinant Cas9 protein (M0386) was purchased from New England Biolabs, Inc. sgRNA were designed by the protocol recommended in Ran et al. [34] Cap1-sgRNA sequence; GCTTGCCGTACAAGCTTGATGG, Lphn2-sgRNA sequence, TACCAGTATATTGCTGCAGTGG. sgRNA was synthesized through in vitro transcription using the MEGAshortscript Transcription Kit (Thermo Fisher Scientific), as described in the manufacturer’s protocols. To assemble the RNP complexes, 300 nM sgRNA was incubated with 300 nM Cas9 nuclease at room temperature for 10 min. Targeting donor vector constructs are purchased from the International Mouse Phenotyping Consortium (IMPC). For zygote injection, 100 ng/uL of linearized donor vector was mixed with RNP and injected to the cytoplasm of mouse zygotes.

### 2.2. Cell Culture

B6/129 F1 hybrid ES (V6.5) cells were cultured in DMEM (Welgene) medium, supplemented with 15% fetal bovine serum (Gibco™, Grand Island, NY), 0.1 mM non-essential amino acids, 4 mM L-glutamine, 0.1 mM 2-mercaptoethanol, 1000 U/mL LIF (Chemicon, Temecula, CA, USA), and 1% penicillin-streptomycin (Gibco™) at 37 °C and with 5% CO_2_, in a humidified incubator. The cells were passaged every 3–4 d with Trypsin-EDTA (Gibco™). The cell lines were quantified with short tandem repeat analysis by KogeneBiotech and tested for mycoplasma contamination every three months, by using the MycoSensor PCR Assay Kit (Agilent). To deliver Cas9 to cells in vitro, cells gat ~80% confluence were treated with an all-in-one vector containing Cas9 and sgRNA or assembled Cas9/sgRNA RNPs, with or without the small molecules (5 μM 5-azacytidine (Sigma), 5 mM VPA (Sigma), 500 nM CTPB (MedChem Express, New Jersey, USA), 25 nM Romidepsin (Selleckchem, Houston, TX, USA), 10 uM SB431542 (R&D Systems), 3 μM CHIR99021 (Peprotech, London, UK), 200 nM Trichostatin A (TSA) (Sigma, St. Louis, MO, USA), or 500 nM Scriptaid (Sigma). The experimenter was not blinded to the treatment. None of the cell cultures were excluded from our analyses.

### 2.3. Mouse Embryo Culture and Microinjection of One-Cell Stage Embryos

All the animal experiments were approved by the Institutional Animal Care and Use Committee at Dongguk University (IACUC-2016-010), and performed in accordance with the institutional guidelines. Mouse embryos were collected from 8-week-old mice with a B6D2F1 (C57BL/6 XDBA2; Jung Ang Lab.Animal Inc., Seoul, Korea) genetic background. Super-ovulated B6D2F1 mice were mated with B6D2F1, and the zygotes were collected from the oviducts. Collected zygotes were cultured with M2 media (#M2101; CytoSpring LLC, Mountain View, CA, USA)), with or without 2 mM of VPA for 1 h. Afterward, one-cell stage mouse embryos were microinjected with pre-assembled RNPs made of Cas9 protein and sgRNA (50 ng/uL) and/or linearized plasmid donor vector (100 ng/μL). The injected one-cell stage embryos were cultured with KSOM (#K0101; CytoSpring LLC), with or without the small molecules at 37 °C and with 5% CO_2_ in a humidified incubator until the blastula stage. Thereafter, mouse blastocysts were analyzed.

### 2.4. Surveyor Assay

We performed the Surveyor nuclease assay using the Surveyor Mutation Detection Kit (Transgenomic, Omaha, NE, USA). Amplified DNA (400 ng) was denatured according to the manufacturer’s instructions. For embryo analysis, we mixed non-microinjected control mouse embryo DNA with the injected samples. Then, the denatured DNA samples were treated with 1 µL of the nuclease and incubated for 30 min at 42 °C. Afterward, the samples were resolved on a 2% TBE agarose gel. Bands were visualized using ImageLab (Bio-Rad Laboratories) and quantitated with the ImageJ (NIH) software.

### 2.5. Western Blot

The cells were detached and homogenized in RIPA buffer (Sigma-Aldrich, St Louis, MO, USA), containing a proteinase inhibitor cocktail (Cell signaling). After homogenization, the samples were mixed with 5X loading buffer (250 mM Tris-HCl, 10% SDS, 30% Glycerol, 500 mM DTT, and 0.05% bromophenol blue) and boiled for 10 min at 100 °C. Proteins were separated with 12% SDS–PAGE and blotted onto a membrane. Blotted membranes were incubated overnight at 4 °C, with an anti-tyrosine hydroxylase antibody (1:1000; P60101-150, Pel-Freez Biologicals). Antibody-reactive bands were then visualized using an enhanced chemiluminscence (ECL) kit (DG-WF200; Dogen). The experimenter was not blinded to the treatment. None of the cell cultures were excluded from analysis.

### 2.6. Sanger Sequencing and PCR Analysis

Amplified PCR products were cloned into a pTOP TA V2 vector with TOPcloner™ TA core Kit (Enzynomics, Seoul, Korea). PCR products in a TA vector were sequenced with M13 sequencing primer (5′-GCG GAT AAC AAT TTC ACA CAG-3′). To detect the HDR-mediated modification, PCR analysis was performed in 20 μL volume containing each specific Cap1 knock-in primer (Forward, CCCCCTGACTCCTTTTCTTC; Reverse, GCAGGGGCTAAATGTGAAAG). Amplified DNA amplicons were separated on 1.5% agarose gel by electrophoresis.

### 2.7. Immunohistochemical Analysis

Embryo samples were collected and fixed with 4% paraformaldehyde. Samples were washed three times with PBS for 5 min each and blocked with 3% BSA blocking solution for 20 min. Then, the samples were incubated with a primary anti-histone H3 (acetyl K27) rabbit antibody (1:250; ab4729, Abcam) overnight at 4 °C. Afterward, the samples were washed three times with PBS for 5 min each. Next, they were incubated with goat anti-rabbit AF-488 (A32731, Invitrogen) secondary antibody for 2 h at room temperature with gentle shaking. Subsequently, the samples were washed three times with PBS for 5 min each and then counterstained with 1 μg/mL 4,6-diamidino-2-phenylindole (1:1000; Invitrogen), at room temperature for 5 min. The samples were examined using a Zeiss LSM 700 confocal microscope (ZEISS). The experimenter was not blinded to the treatment. None of the cell cultures were excluded from analysis.

### 2.8. Bioinformatics Analysis

For chromatin immunoprecipitation and sequencing (ChIP-Seq) analysis, we accessed published H3K9ac ChIP data produced by high-throughput sequencing from mouse embryonic stem cells before (control) and after 16 h of VPA treatment [35,36]. We aligned raw sequencing data to the mouse genome using Bowtie2 with two groups of embryonic stem cells, with and without VPA treatment of 16 h. Then, using the HOMER and Integrative Genomics Viewer (IGV) tools, the peaks of H3K9 acetylation and the distribution of fragment depth were shown. All sequencing data were downloaded from the Gene Expression Omnibus (GEO) data store by accession number GSM595515, GSM595517, GSM595518. Off-target sites of Cas9 RNA-guided endonucleases were detected by Cas-OFFinder software, which is a highly versatile off-target searching tool (http://www.rgenome.net/cas-offinder/). Predicted off-target sites identified being confirmed by Surveyor assay.

### 2.9. Statistical Analysis

All data are presented as mean ± standard deviation of three independent experiments. Statistical analyses were performed with SPSS version 18.0 (IBM Corporation). Group differences were considered statistically significant in * *p* < 0.05 and ** *p* < 0.01. Significant differences between groups were analyzed with a one-way analysis of variance (ANOVA). Data were normally distributed and analyzed by two-tailed Student’s *t*-test, and statistical significance was determined at *p* < 0.05. The image analysis of samples were blinded and performed by independent investigators. Data collection and analyses were performed blinded and were randomized.

## 3. Results

### 3.1. Effect of Valproic Acid (VPA) on CRISPR/Cas9-Mediated Gene Targeting In Vitro

**Figure 1:**
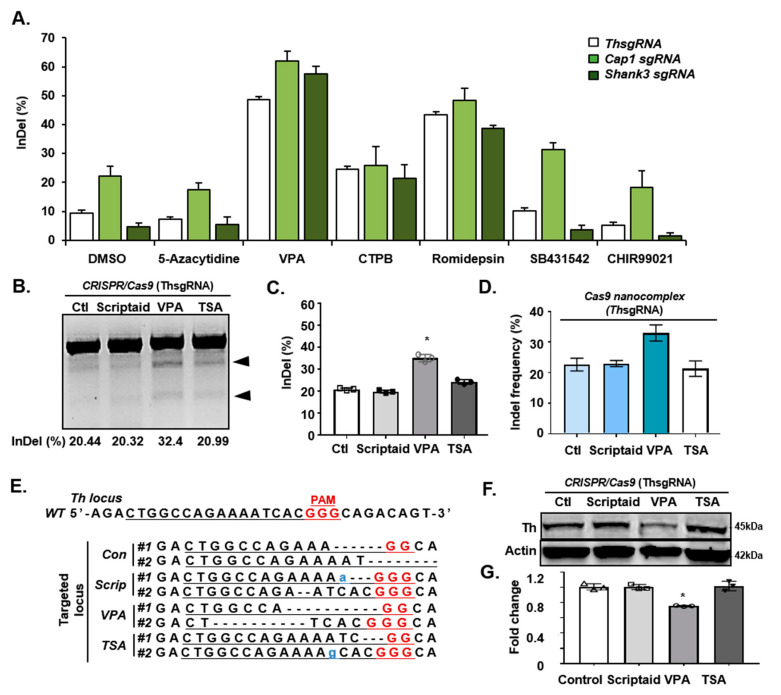
Valproic acid (VPA) enhances clustered regularly interspaced short palindromic repeats (CRISPR)/Cas9-mediated in vitro targeting efficiency. (**A**) Percentage of InDel frequencies, according to the Surveyor assay results. The assay was performed with various sgRNAs targeting Th, Cap1, and Shank3 genes in mESCs in the presence of various small molecules (DMSO, 5-azacytidine, VPA, CTPB, Romidepsin, SB431542, or CHIR99021), by transfection with dual Cas9 and sgRNA vector. (**B**) The Surveyor assay in mESCs co-treated with Scriptaid, VPA, or Trichostatin A (TSA) and targeted for Th. Ctl, control. (**C**) Percentage of the InDel frequencies according to the Surveyor assay results. Data are expressed as mean ± SD, *n* = 3. * *p* < 0.05, one-way analysis of variance (ANOVA) with Tukey’s post-hoc test. (**D**) The InDel frequencies on Th gene identified by sequencing of the mESCs co-treated with scriptaid, VPA, or TSA. (**E**) Sanger sequencing analysis of the Th locus in mESCs co-treated with Scriptaid, VPA, or TSA. Red, PAM sequence; Underline, guide sequence. Scrip, Scriptaid. (**F**) Western blot showing the effect of scriptaid, VPA, or TSA co-treatment in Th protein levels in mESC-derived dopaminergic neurons. (**G**) Quantification of the western blot analysis in Figure 1F. Data are expressed as mean ± SD, *n* = 3. * *p* < 0.05, one-way ANOVA with Tukey’s post-hoc test. The images in B and F are each representatives of ≥3 similar experiments.

Previous studies have shown that HDAC inhibitors significantly enhance the efficiency of cell fate conversions by affecting the chromatin structure [37,38]. Likewise, we speculated that the efficiency of CRISPR/Cas9-mediated gene editing may be increased when the chromatin structure is opened by the treatment of HDAC inhibitors. Accordingly, we tested whether chromatin-modifying small molecules had any effect on CRISPR/Cas9-mediated gene targeting. For proof of concept, we selected different genes, such as tyrosine hydroxylase (Th), cyclase-associated actin cytoskeleton regulatory protein 1 (Cap1), or SH3 and multiple ankyrin repeat domains protein 3 (Shank3) that are expressed in various cell types, to see their effects on various cells. We found that CRISPR/Cas9 targeting of these genes induced approximately 10–20% of InDels in mouse ESCs (mESCs) by day 4 of CRISPR/Cas9 treatment. Previously, since it was known that these small molecules influenced the structure of chromatin or gene expression [39,40,41,42,43,44], we selected molecules and tested the gene targeting efficiency. Treatment of the cells with 5-azacytidine (DNA demethylation inhibitor), CHIR99021 (GSK-3 Inhibitor), SB431542 (TGF-beta receptor inhibitor), or CTBP (transcriptional repressor) had no significant effects on the efficiency of gene targeting (Figure 1A). However, Romidepsin as HDAC inhibitor treatment increased the gene targeting efficiency by approximately two-fold to ~45%. Strikingly, we found that treating CRISPR/Cas9-transduced mESCs with 5 mM VPA for 4 d induced 60–70% of gene targeting, amounting to a >6-fold improvement over the control (Figure 1A). Additionally, we tested several other HDAC inhibitors, such as Scriptaid and TSA, in comparison to VPA. Consistent with the previous result, VPA had the most potent enhancer activity in CRISPR/Cas9 gene targeting (Figure 1B–G). Next, the surveyor assay was performed to evaluate the targeting efficiency. The results showed that VPA treatment of mESCs yielded the highest InDels percentage (Figure 1B,C, Figure S1A,B). Moreover, Sanger sequencing of the targeted *Th* locus confirmed the nonhomologous end-joining (NHEJ)-induced InDels in the cells treated with VPA (Figure 1D,E). We also confirmed that the CRISPR/Cas9 targeting of Th significantly downregulated Th expression in ESC-derived dopaminergic neurons (Figure 1F,G). Taken together, these data show that efficient CRISPR/Cas9-mediated gene targeting can be achieved with VPA treatment in mESCs.

### 3.2. Effect of VPA on CRISPR/Cas9-Mediated NHEJ in Mouse Embryos

**Figure 2:**
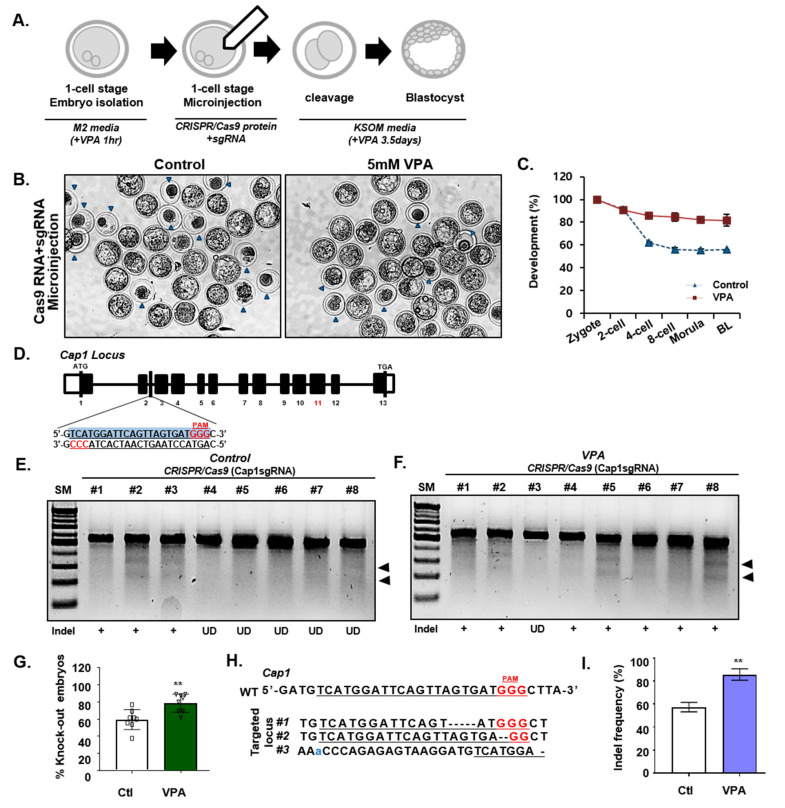
VPA enhances CRISPR/Cas9-mediated knock-out efficiency in mouse embryos. (**A**) Schematic representation of CRISPR/Cas9 targeting in mouse embryos. Fertilized zygotes were cultured in M2 media, with or without VPA treatment for an hour, and microinjected with pre-assembled CRISPR/Cas9 RNP complexes. Afterward, the zygotes were cultured in KSOM media, with or without VPA for 3.5 days. (**B**) Images of the control or VPA-treated (5 mM) mouse blastocysts. Blue arrows indicate the embryos that failed to form blastocysts. (**C**) Percentage of viable mouse embryos in the control or VPA-treated groups at each developmental stage. (**D**) Schematic illustration depicting the targeting strategy for Cap1 locus. The targeting sequence is underlined, and PAM sequence is in red font. (**E**,**F**) The Surveyor assay for the InDel mutations induced by Cap1sgRNA (**E**) without or (**F**) with VPA-treatment in mouse embryos. The arrow indicates Surveyor-nuclease–digested PCR products. (**G**) The knock-out efficiency, without or with VPA-treatment. Data were expressed as mean ± SD, *n* = 3. ** *p* < 0.01, two-sided Student’s *t*-test. (**H**) Sanger sequencing analysis of the Cap1 locus in the embryos CRISPR/Cas9-targeted, with or without VPA-treatment. The sgRNA sequence is underlined, and PAM sequence is in red font. (**I**) The InDel frequencies in Cap1 determined by Sanger sequencing in mouse embryos CRISPR/Cas9-targeted, without or with VPA-treatment. Data were expressed as mean ± SD, *n* = 3. ** *p* < 0.01, two-sided Student’s *t*-test. The images in B, E, and F are each representatives of ≥ 3 similar experiments.

Next, we evaluated the efficacy of the CRISPR/Cas9 system in one-cell stage mouse embryos. Cas9/sgRNA ribonucleoproteins (RNPs) targeting Cap1 or Lphn2 were injected into the embryos and the targeting efficiencies were assessed during the blastocyst stage, and were evaluated according to the presence or absence of VPA treatment (Figure 2A). We found that the number of mature blastocysts indicated that VPA treatment improves the development of embryos into the blastocysts stage, a pattern similar to the results of VPA treatment previously reported [45,46,47]. (Figure 2B,C, Figure S2A,B, and Table S1). However, the blastomere number in cleavage-stage embryos was not associated with VPA treatment (Figure S2C,D). Moreover, the Surveyor assay on day 3.5 post-injection of the sgRNA/Cas9 RNPs revealed efficient targeting of the Cap1 locus in mouse embryos (Figure 2D–F), which comprised a significant number of InDels of Cap1 and Lphn2 alleles (Figure 2G, and Table 1). Sanger sequencing of the targeted region also identified the InDels caused by NHEJ in the Cap1 coding sequence and their frequency (Figure 2H,I). Finally, we confirmed the off-target effects in the VPA mediated efficient gene targeting. The Cap1 off-targets were selected from the ‘Cas OFFinder’ software and examined the off-targets by Surveyor assay. We found that Cap1sgRNA-induced off-target mutations captured by ‘Cas-OFFinder’ were not detected in the control or VPA-treatment groups (Figure S3A,B), suggesting the VPA effect on CRISPR/Cas9-mediated on-target gene targeting in mouse embryos.

### 3.3. Effect of VPA on CRISPR/Cas9-Mediated HDR in Mouse Embryos

**Figure 3:**
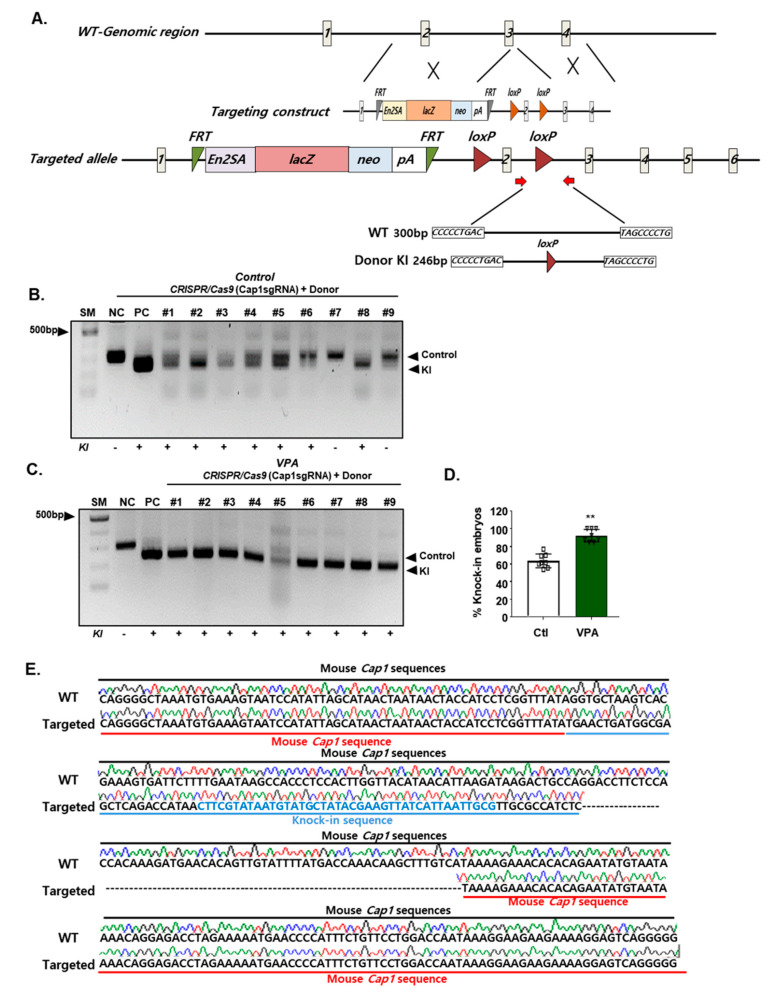
VPA enhances CRISPR/Cas9-mediated knock-in efficiency in mouse embryos. (**A**) Schematic illustration depicting the targeting strategy for the Cap1 locus. Primers used for PCR analysis are represented by red arrows. En2SA: splice acceptor site of exon 2 of the mouse engrailed-2 gene. LacZ: β-galactosidase; Neo: neomycin-resistance. (**B**) Representative separation of Cap1 PCR products in the mouse embryos CRISPR/Cas9-targeted without VPA-treatment after 3.5 days of injection. The upper and lower arrows indicate the PCR fragments without (300 bp) and with (246 bp) Cap1 knock-in donors, respectively. (**C**) Representative of Cap1 PCR products in the mouse embryos CRISPR/Cas9-targeted with VPA-treatment. (**D**) The knock-in efficiency in the mouse embryos evaluated using PCR. Data were expressed as mean ± SD, *n* = 3. ** *p* < 0.01, two-sided Student’s *t*-test. (**E**) Sanger sequencing of knock-in mouse Cap1 locus. Blue colored sequences are loxP knock-in sequences. The images in B and C are representatives of ≥ 3 similar experiments.

Next, we examined whether efficient homology-mediated recombination of a transgene into an endogenous locus can be mediated by VPA-augmented CRISPR/Cas9 targeting. PCR analysis of the donor DNA showed that VPA treatment significantly improved the knock-in efficiency in mESCs (Figure S1B); moreover, CRISPR/Cas9 gene editing in mouse blastocysts treated with VPA showed up to approximately 100%, whereas sgRNA/Cas9 proteins without VPA showed a knock-in efficiency of 60% (Figure 3A–D, and Table 2). Furthermore, we confirmed the integration of the donor DNA at the mouse Cap1 locus by Sanger sequencing (Figure 3E). These results demonstrate the efficient CRISPR/Cas9 gene editing in mouse blastocysts treated with VPA.

### 3.4. Mechanism of VPA in the CRISPR/Cas9-Based Efficient Gene Editing

**Figure 4:**
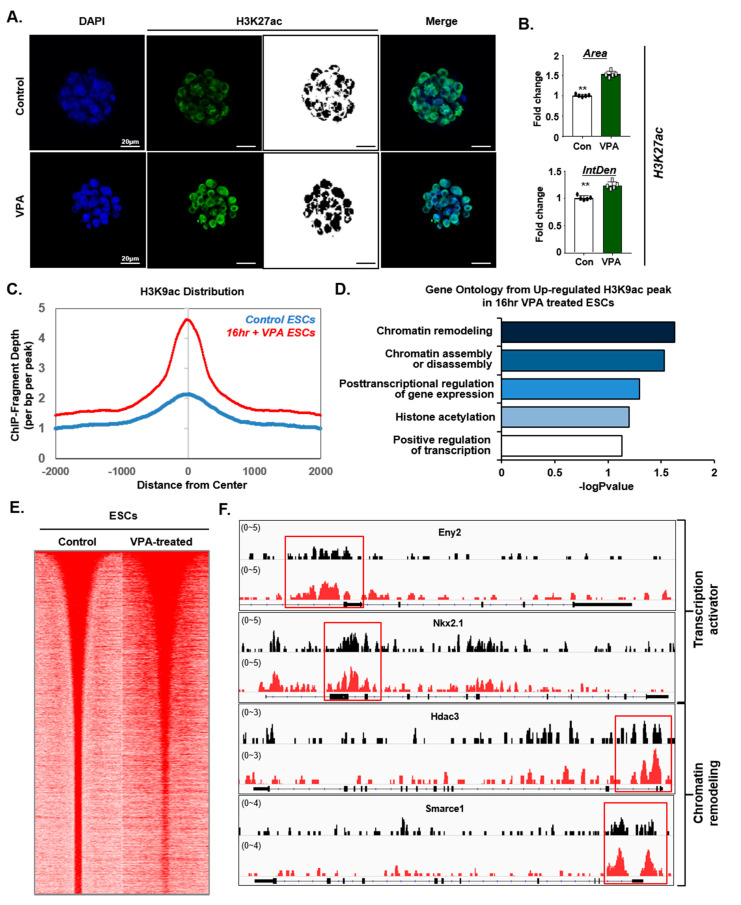
VPA increases acetylation and occupancy on pluripotent genes. (**A**) Immunofluorescence of H3K27ac in mouse blastocysts before (control) or after VPA-treatment. (**B**) Quantification of the H3K27ac-positive area and intensity in mouse blastocysts. Data were expressed as mean ± SD, *n* = 5. ** *p* < 0.01, two-sided Student’s *t*-test. (**C**) H3K9ac ChIP-seq enrichment occupancies of the control samples or those treated with VPA for 16 h. Enrichments were plotted ±2 kb around the TSS in mESCs. Y-axis represents the depth of ChIP-fragment. (**D**) Gene ontology (GO) enrichment analysis of the H3K9ac upregulated genes, with 16 h of VPA-treatment in mESCs. (**E**) Heatmaps represent relative ±2 kb around H3K27ac peak levels from mouse ESCs whole-genome before (control) or after VPA-treatment. (**F**) Genome browser tracks show the occupancy of transcription activator and chromatin remodeling near Eny2, Nkx2.1, Hdac3, and Smarce1 loci. The image in A is representative of ≥3 similar experiments.

To better understand the effect of VPA on the enhancement of CRISPR/Cas9-mediated gene editing, we compared the level of histone acetylation (H3K27ac) in CRISPR/Cas9-targeted mESCs, in the presence and absence of VPA. Consistent with these results and drawing on publicly available, previously published data [35,36], we found that the average intensity of histone acetylation (H3K27ac) was significantly higher in the VPA-treated group than in the control group, whereas the *H3K27me3* signal decreased (Figure 4A,B, Figure S4A,B). Consistent with these results, we found that the H3K9 acetylation at the promoter regions was highly increased in the VPA-treated mESCs (Figure 4C). Additionally, gene ontology enrichment analysis showed that the genes with H3K9ac increases were related to chromatin remodeling, chromatin assembly, regulation of gene expression, histone acetylation, and regulation of transcription (Figure 4D). Moreover, H3K27ac displayed strong enrichment around the TSS region in the VPA-treated mESCs (Figure 4E). The individual gene tracks confirmed the increased occupancy of transcription activator and chromatin remodeling complexes on pluripotency genes, such as Eny2, Nkx2.1, Hdac3, and Smarce1 (Figure 4F), suggesting globally enhanced chromatin accessibility by VPA treatment.

## 4. Discussion

CRISPR/Cas9-mediated precise gene targeting has great promise in the gene therapy of many diseases and transgenic applications. However, the low efficiency of CRISPR/Cas9-mediated gene editing still hinders the application of this technology in various biomedical applications. This study demonstrated that the highly efficient Cas9-mediated gene editing of mESCs and mouse embryos can be achieved by co-treatment with the HDAC inhibitor VPA. Furthermore, we reasoned that this effect is produced through the open chromatin structure in mouse ESC and embryos. Thus, our data vindicate the biochemical approach involving a chromatin modifier VPA for highly efficient CRISPR/Cas9-mediated transgenic applications.

We selected six small molecules based on previous studies and found that HDAC inhibitors enabled efficient gene editing in mESCs. To further identify the small molecules that led to the most efficient gene editing, several HDAC inhibitors were individually examined in mESCs. We found that co-treatment of the cells with the HDAC inhibitor VPA resulted in the most efficient gene editing. VPA is used for the treatment of various psychiatric diseases, such as epilepsy and bipolar mania [30,48,49,50]. It is also widely used in cellular reprogramming studies to eliminate the original epigenetic memory of differentiated cells, thereby promoting the pluripotent activity [31] in human and mouse cells [51]. Several lines of evidence suggest that VPA enhances reprogramming efficiency through several mechanisms, including open chromatin structure [31,52]. Consistent with these studies, we observed that VPA significantly improved CRISPR/Cas9-mediated gene editing efficiency, possibly mediated through modified chromatin structure.

In addition to the enhanced gene editing efficiency, the advantage of using VPA is that modulating the treatment dosage and period may enable temporal control of gene editing. Thus, this chemical-augmented approach may provide a safer and more precise gene editing in therapeutic and transgenic applications. Furthermore, since VPA can stimulate the differentiation and development of mouse embryos, it is expected to have a more positive effect on the generation of transgenic animals. However, it is currently unclear whether all genes acetylated upon VPA treatment can efficiently be targeted by CRISPR/Cas9, and we aim to elaborate on this in future studies.

Finally, our studies provide evidence of the principle that chemical-augmented approaches in the CRISPR/Cas9 mediated gene editing which may be useful for on the generation of transgenic animals, in addition to the established potential benefit of VPA treatment for safer and more accurate gene editing in therapeutic applications.

## Figures and Tables

**Table 1:** Efficient CRISPR/Cas9-mediated gene targeting in mouse embryos. Cas9 protein and sgRNA targeting Cap1 or Lphn2 were injected into the cytoplasm of mouse embryos. The zygotes were cultured with or without VPA in M2 media 1 h before the microinjection, and cultured in KSOM medium with or without VPA until the blastocysts stage (3.5 days of culture) and analyzed by PCR and Sanger sequencing.

Treatment	Targeting System	Blastocysts /Injected Zygotes	Targeted Blastocysts/Total	InDel Mutation Efficiency (%)
Control	Cas9 protein + Cap1 sgRNA	34/60	21/34	61.8
VPA	Cas9 protein + Cap1 sgRNA	43/50	32/43	74.4
VPA	Cas9 protein + Cap1 sgRNA	45/50	36/45	80
Control	Cas9 protein + Lphn2 sgRNA	35/60	20/35	57.1
VPA	Cas9 protein + Lphn2 sgRNA	53/60	49/53	92.5
VPA	Cas9 protein + Lphn2 sgRNA	55/60	48/55	87.3

**Table 2:** CRISPR/Cas9-mediated knock-in efficiencies in mouse embryos. Cas9 protein, Cap1 sgRNA, and the linearized Cap1 donor vector were injected into the cytoplasm of the mouse embryos. The zygotes were cultured with or without VPA in M2 media 1 h before the microinjection and cultured in KSOM medium, with or without VPA until the blastocysts stage (3.5 days of culture) and analyzed by PCR and Sanger sequencing.

Treatment	Targeting System	Blastocysts /Injected Zygotes	Targeted Blastocysts/Total	Knock-In Efficiency (%)
Control	Cas9 protein + Cap1 sgRNA + Cap1 Donor	29/55	18/29	62.1
Control	Cas9 protein + Cap1 sgRNA + Cap1 Donor	25/50	25/39	64.1
VPA	Cas9 protein + Cap1 sgRNA + Cap1 Donor	32/40	32/34	94.1
VPA	Cas9 protein + Cap1 sgRNA + Cap1 Donor	45/55	45/50	90

## Supplementary Materials

The following are available online at https://www.mdpi.com/2073-4409/9/6/1447/s1, Figure S1: VPA enhances CRISPR/Cas9-mediated targeting efficiency; Figure S2: Effect of VPA on embryonic development; Figure S3: Surveyor assay for potential Cap1 off-target sites; Figure S4: Distribution of tri-methylated H3K27 in VPA-treated or untreated mouse embryo; Figure S5: Full scans of the western blotand DNA gel presented in this study. Rectangles delimit cropped area used in the figure; Table S1: Effects of VPA on the development of CRISPR/Cas9 injected embryo under different concentration.
