# Supplementary material for: Valproic Acid Significantly Improves CRISPR/Cas9-Mediated Gene Editing
Source: Cells. 2020 Jun 10;9(6):1447. doi: 10.3390/cells9061447 (PMC7349485; doi:10.3390/cells9061447)
Supplement: Supplementary file 1 [file cells-09-01447-s001.pdf]

Figure S1

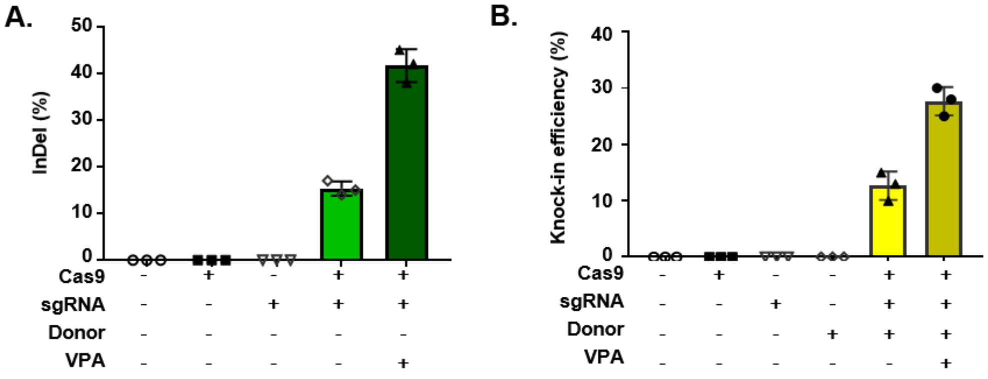

Figure 1. VPA enhances CRISPR/Cas9-mediated targeting efficiency. (**A**) The percentage of InDel efficiency in mouse embryonic stem cells evaluated using sequencing analysis. Data are expressed as mean ± SD, $n = 3$. *$p < 0.05$, one-way analysis of variance (ANOVA) with Tukey's post hoc test. (**B**) The knock-in efficiency in the mouse embryonic stem cells evaluated using sequencing analysis. Data are expressed as mean ± SD, $n = 3$. *$p < 0.05$, one-way analysis of variance (ANOVA) with Tukey's post hoc test.

Figure S2

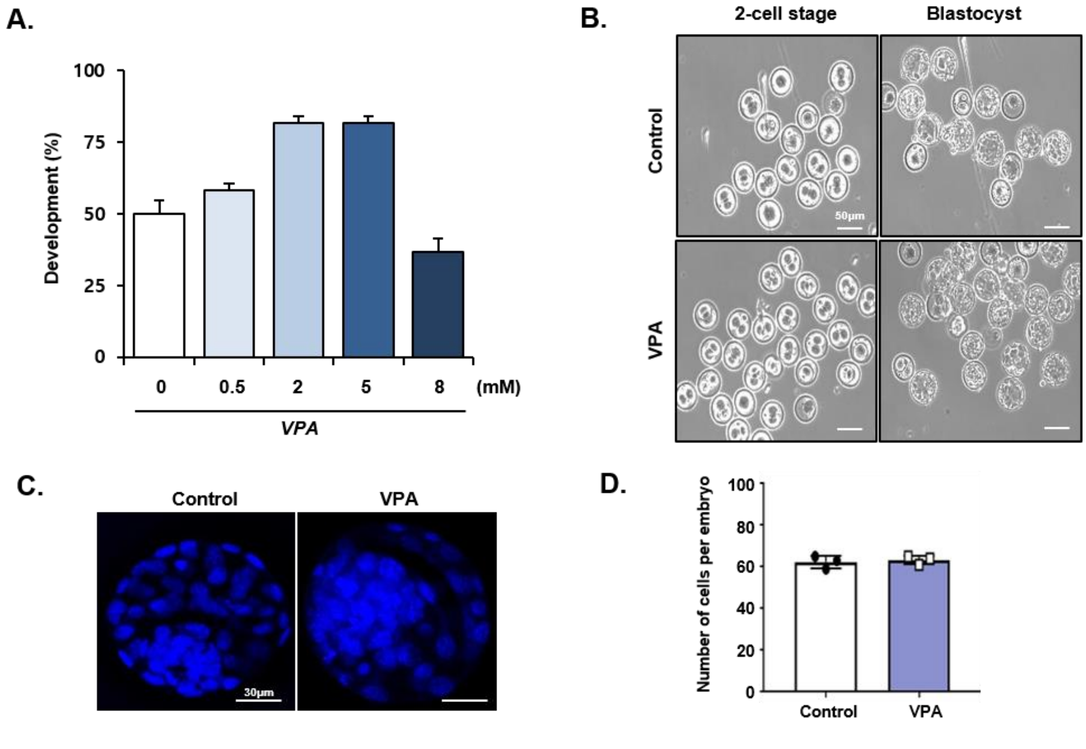

Figure 2. Effect of VPA on embryonic development. (**A**) Percentage of blastocysts (early embryonic development) formation of mouse embryo treated with various concentrations of VPA (**B**) Morphology of the embryos untreated or treated with VPA. Morphologies are at different embryonic stages are shown. (**C**) Mouse blastocysts were stained with DAPI to visualize DNA (blue). (**D**) Average cell numbers of blastocysts treated or untreated with VPA. The images in B and C are representatives of ≥ 3 similar experiments.

Figure S3

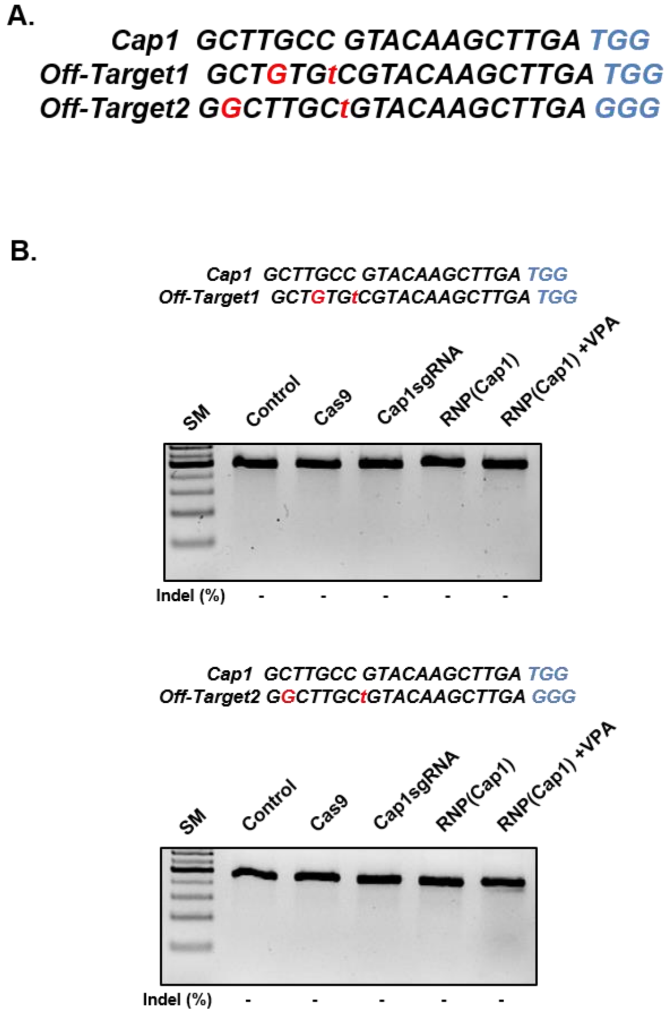

Figure 3. Surveyor assay for potential Cap1 off-target sites. (**A**) Top2 predicted off-target sites for Cap1 sgRNA sequence by Cas-OFFinder software. (**B**) Surveyor assay for the InDel mutations induced by Cap1 sgRNA in top2 Cap1 off-target sites.

Figure S4

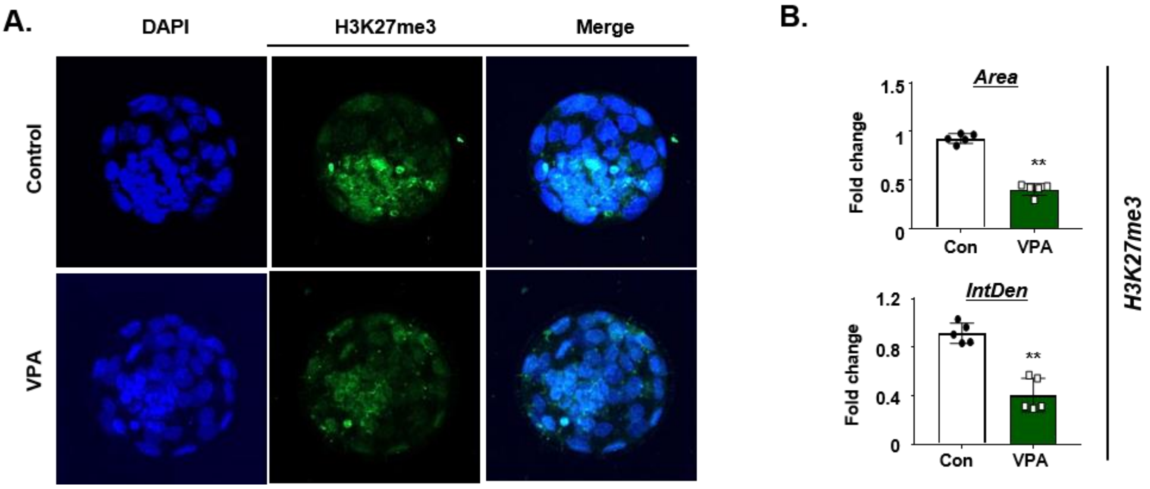

Figure 4. Distribution of tri-methylated H3K27 in VPA-treated or untreated mouse embryo. (**A**) Immunofluorescence of H3K27me3 in mouse blastocysts with or without VPA-treatment. (**B**)

Quantification of the H3K27me3-positive area and intensity in the mouse blastocysts. Data are expressed as mean ± SD, $n = 5$. $^{**}p < 0.01$, two-sided Student's t-test. The images in a is representatives of ≥ 3 similar experiments.

Figure S5

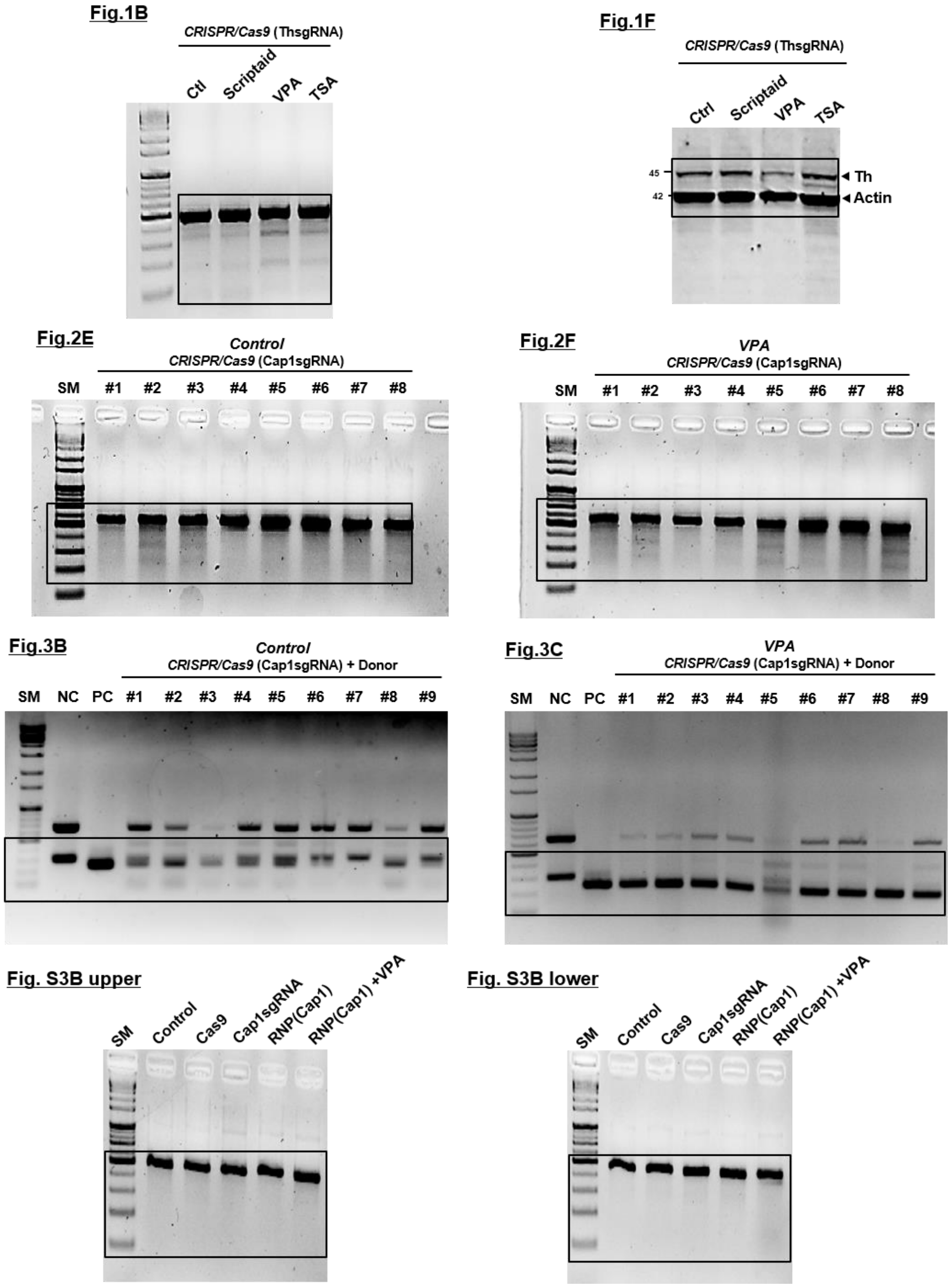

Figure 5. Full scans of the western blotand DNA gel presented in this study. Rectangles delimit cropped area used in the figure.

Table 1. Effects of VPA on the development of CRISPR/Cas9 injected embryo under different concentration.

		No. of Embryos Developed to (%)	
Concentration	No. of injected oocytes	Cleavage	Blastocyst
0 mM	60	55 (91.6)	30 (50)

0.5 mM	60	56 (93.3)	35 (58.3)
2 mM	60	54 (90)	49 (81.6)
5 mM	60	53(88.3)	49 (81.6)
8 mM	60	56 (93.3)	22 (36.6)
